# Clinical use of mean nocturnal baseline impedance and post-reflux swallow-induced peristaltic wave index for the diagnosis of gastro-esophageal reflux disease

**DOI:** 10.1007/s10388-022-00933-6

**Published:** 2022-06-29

**Authors:** Pierfrancesco Visaggi, Lucia Mariani, Federica Baiano Svizzero, Luca Tarducci, Andrea Sostilio, Marzio Frazzoni, Salvatore Tolone, Roberto Penagini, Leonardo Frazzoni, Linda Ceccarelli, Vincenzo Savarino, Massimo Bellini, Prakash C. Gyawali, Edoardo V. Savarino, Nicola de Bortoli

**Affiliations:** 1 Division of Gastroenterology, Department of Translational Research and New Technologies in Medicine and Surgery, School of Medicine, University of Pisa, Pisa, Italy; 2 Digestive Pathophysiology Unit, Baggiovara Hospital, Modena, Italy; 3 General and Bariatric Surgery Unit, Department of Surgery, University of Caserta Luigi Vanvitelli, Caserta, Italy; 4 Department of Pathophysiology and Transplantation, University of Milan, Milan, Italy; 5 Gastroenterology and Endoscopy Unit, Fondazione IRCCS Ca’ Granda Ospedale Maggiore Policlinico, Milan, Italy; 6 Gastroenterology Unit, Department of Medical and Surgical Sciences, Sant’Orsola Hospital, University of Bologna, Bologna, Italy; 7 Gastroenterology Unit, Department of Internal Medicine “DiMI”, University of Genoa, Genoa, Italy; 8 Division of Gastroenterology, Washington University School of Medicine, St. Louis, USA; 9 Division of Gastroenterology, Department of Surgical, Oncological and Gastroenterological Sciences, University of Padua, Padua, Italy

**Keywords:** MNBI, PSPW index, GERD, Diagnosis, Reflux disease

## Abstract

The clinical diagnosis of gastro-esophageal reflux disease (GERD) is based on the presence of typical esophageal troublesome symptoms. In clinical practice, heartburn relief following a proton pump inhibitor (PPI) trial or endoscopy can confirm a diagnosis of GERD. In cases of diagnostic uncertainty or before anti-reflux interventions, combined impedance-pH monitoring (MII-pH) provides a comprehensive assessment of both physical and chemical properties of the refluxate, allowing to achieve a conclusive diagnosis of GERD. Recently, the Lyon Consensus proposed the use of mean nocturnal baseline impedance (MNBI) and post-reflux swallow-induced peristaltic wave index (PSPW-I) as novel MII-pH metrics to support the diagnosis of GERD. The calculation of MNBI and PSPW-I currently needs to be performed manually, but artificial intelligence systems for the automated analysis of MII-pH tracings are being developed. Several studies demonstrated the increased diagnostic yield MNBI and PSPW-I for the categorization of patients with GERD at both on- and off-PPI MII-pH monitoring. Accordingly, we performed a narrative review on the clinical use and diagnostic yield of MNBI and PSPW-I when the diagnosis of GERD is uncertain. Based on currently available evidence, we strongly support the evaluation of PSPW-I and MNBI as part of the standard assessment of MII-pH tracings for the evaluation of GERD, especially in patients with endoscopy-negative heartburn.

## Introduction

Gastro-esophageal reflux disease (GERD) is one of the most prevalent gastrointestinal disorders and represents a risk factor for Barrett’s esophagus (BE) and esophageal adenocarcinoma [1, 2]. GERD consists of troublesome symptoms or mucosal damage resulting from retrograde movement of the gastric content through an incompetent esophagogastric junction (EGJ) [1]. The prevalence of GERD based on symptom perception in individual cross-sectional surveys varies from 2.5% to more than 25% [3–7], depending on the criteria used to define their presence and frequency, and the geographical location of the study, with lower rates in Asia compared to Western countries [8, 9].

The pathophysiology of GERD is complex and involves several different mechanisms, including impairment of esophageal inherent protective mechanisms (e.g., intact reflux-induced swallow and secondary peristalsis), disruption of the EGJ, delayed gastric emptying, hypersecretory states, or reflux hypersensitivity (RH) [10]. In this regard, there is evidence that suboptimal esophageal clearance, impaired esophageal mucosal defense, abnormalities of the lower esophageal sphincter (LES), frequent transient LES relaxations (TLESRs), and a reduced LES pressure, synergistically contribute to the development of GERD [11, 12].

A clinical diagnosis of GERD is suspected based on the presence of typical symptoms (i.e., heartburn and regurgitation) and can be subsequently confirmed by symptoms improvement following treatment with proton pump inhibitors (PPIs) or suggestive findings on esophagogastroduodenoscopy (EGDS) [13–15]. Additionally, GERD-specific questionnaires may support the diagnosis of GERD [16]. In this regard, a recent meta-analysis found that novel artificial intelligence (AI) systems had sensitivity and specificity of 97% with an area under the receiver operating characteristic curve of 99% for the diagnosis of GERD based on questionnaires [17]. The presence of alarm symptoms (e.g., dysphagia, weight loss, anemia), atypical presentations (e.g., chest pain, laryngeal symptoms) or lack of response to empiric therapy, prompt further evaluation with an EGDS [13, 18]. If symptoms persist despite empiric therapy and the EGDS does not reveal objective evidence of GERD (e.g., esophagitis, esophageal peptic stricture, BE), esophageal function tests are subsequently performed, including esophageal manometry and ambulatory reflux monitoring [19].

In recent years, the use of ambulatory reflux monitoring with impedance allowed a more sophisticated analysis of esophageal physiology and provided novel insights into GERD sub-types. In this regard, up to 70% of patients with esophageal symptoms suggestive of GERD are found to have a normal EGDS and are, therefore, categorized as having non-erosive reflux disease (NERD) [20–22]. However, NERD is an “umbrella term” which includes heterogeneous subpopulations [23]. Most patients with endoscopy-negative heartburn are classified as NERD based on abnormal acid exposure at pH or impedance-pH (MII-pH) monitoring [23, 24], others have RH based on normal EGDS, normal reflux monitoring, and a positive correlation between reflux episodes and symptoms occurrence at MII-pH [25]. Patients with normal EGDS and normal reflux monitoring without reflux–symptom correlation are diagnosed with functional heartburn (FH), which is considered a separate entity from GERD [26].

Recently, the Lyon Consensus discussed the performance characteristics of available diagnostic strategies for a modern diagnosis of GERD, including recently introduced MII-pH metrics, namely mean nocturnal baseline impedance (MNBI) and post-reflux swallow-induced peristaltic wave (PSPW) index [27]. Accordingly, the aim of this narrative review was to summarize the most recent literature on the clinical use of MNBI and PSPW-I according to the Lyon Consensus and to provide updated evidence on the utility of these novel MII-pH parameters for a conclusive diagnosis of GERD.

### Modern diagnosis of GERD: the Lyon Consensus

According to the Montreal definition, GERD is defined by the occurrence of heartburn at least twice weekly, although the disease may present with severe and less frequent symptoms in some patients [1, 21, 23]. The sensitivity and specificity of a GERD diagnosis based on symptoms assessed by gastroenterology specialists are 67% and 70%, respectively. Of note, the Diamond Study found that the diagnostic performance of gastroenterologists was comparable to that of family practitioners and the reflux disease questionnaire (RDQ) [16]. More recently, a meta-analysis conducted by Visaggi et al. estimated a diagnostic accuracy close to 100% for the diagnosis of GERD based on questionnaires when AI systems are used to evaluate symptoms [17].

Although the resolution of symptoms following a 2-week PPI trial in patients with clinically suspected GERD occurs in half of patients, as much as 35% of those without GERD may experience symptoms improvement [16]. Accordingly, a positive response to an empiric treatment with PPIs has modest accuracy for the diagnosis of GERD. However, the sensitivity and positive predictive value (PPV) of a PPI trial may reach 71% and 84%, respectively, in patients who report typical symptoms as their most troublesome [16].

In patients with alarm symptoms, or in those whose response to PPIs is insufficient, an EGDS should be performed after two to three weeks from PPI discontinuation, which will allow to rule out the presence of erosive esophagitis, BE, peptic strictures, or eosinophilic esophagitis [28–30]. When endoscopy rules out macroscopic alterations but patients complain of GERD symptoms, a diagnosis of NERD, RH, or FH is possible [26]. In such instances, reflux monitoring is indicated [27]. Ambulatory reflux monitoring is useful to assess GERD in case of diagnostic uncertainty, in those with PPI-refractory symptoms, when presenting symptoms are atypical, or prior to invasive anti-reflux therapy [31]. Although pH monitoring provides information on the acid exposure time (AET) of the esophagus (pH < 4), the assessment of weakly acidic (pH 4–7) and alkaline refluxes (pH > 7) and bolus exposure requires MII-pH evaluation [31–34]. Accordingly, the addition of impedance to ambulatory pH monitoring increases the sensitivity and specificity of the test for the identification of reflux episodes [35]. With regard to AET, the Lyon Consensus proposed that an AET < 4% should be considered normal and an AET > 6% conclusively abnormal. Similarly, the presence of < 40 reflux episodes per 24 h is considered physiological, while > 80 is abnormal [27].

Of particular note, when the AET is between 4 and 6%, the diagnosis of GERD is inconclusive, and the evaluation of adjunctive parameters should be taken into account to achieve a conclusive diagnosis [27]. In this regard, several metrics might be helpful, including the symptom index (SI) and the symptom association probability (SAP). SI and SAP are two metrics used to estimate the reflux–symptom association. The SI reflects the number of symptom events preceded by a reflux episode during a 24-h MII-pH. When the proportion is > 50%, the SI is considered positive [36]. The SAP indicates the probability that reflux episodes are associated with the occurrence of symptoms [37, 38]; the SAP is considered positive when > 95%. The combination of a positive SI and SAP provides evidence of a clinically relevant association between reflux symptoms and episodes, which could predict treatment response [39, 40].

More recently, the application of high-resolution esophageal manometry (HRM) in the setting of GERD was evaluated. In this regard, HRM is helpful to assess the characteristics of the EGJ and to rule out peristaltic disorders as possible triggers of esophageal symptoms [41, 42]. Additionally, a type 3 morphology of the EGJ (i.e., separation between LES and crural diaphragm ≥ 3 cm) at HRM could support the diagnosis of GERD [27, 43]. Accordingly, the incompetence of the EGJ impairs the anti-reflux barrier and represents a mechanism of GERD [27, 44, 45]. The fourth iteration of the Chicago Classification [46] recently proposed the EGJ contractile integral (EGJ-CI) as a parameter to define the lack of efficacy of the EGJ barrier. The EGJ-CI is the integral of the contractile vigor of the EGJ and has been shown to identify patients with severe EGJ barrier dysfunction [47]. Additionally, the assessment of the peristaltic vigor of the esophageal body might be useful in the diagnosis of GERD [27, 48]. In this regard, ineffective esophageal motility is associated with higher acid burden. However, the relationship between esophageal contractile vigor and acid burden is not univocal as the reduction in contractile vigor increases the likelihood of higher AET and chronic GERD causes esophageal motor dysfunction [49, 50].

### Mean nocturnal baseline impedance

Impedance is defined as the opposition to electrical current within a closed circuit and can be considered as analogous to resistance [51]. The exposure of the esophageal mucosa to noxious agents and subsequent mucosal damage cause a reduction of transepithelial electrical resistance [52]. Accordingly, esophageal impedance is an indicator of mucosal integrity and reflects the modifications of the permeability of the esophageal epithelium, which are primarily due to the dilation of intercellular spaces and the disruption of tight junctions, even when macroscopic changes are absent [53, 54]. The value of esophageal impedance reduces during the exposure to a liquid bolus and increases in the presence of air. Accordingly, the measurement of the mucosal impedance of the esophagus during MII-pH can distinguish the composition and the direction (anterograde or retrograde) of intraluminal contents [55].

Conventional MII-pH parameters may have suboptimal sensitivity for the diagnosis of GERD [54]. Recently, MNBI and PSPW index (PSPW-I) have been proposed as novel MII-pH parameters to increase the diagnostic yield of ambulatory reflux monitoring.

**Fig. 1:**
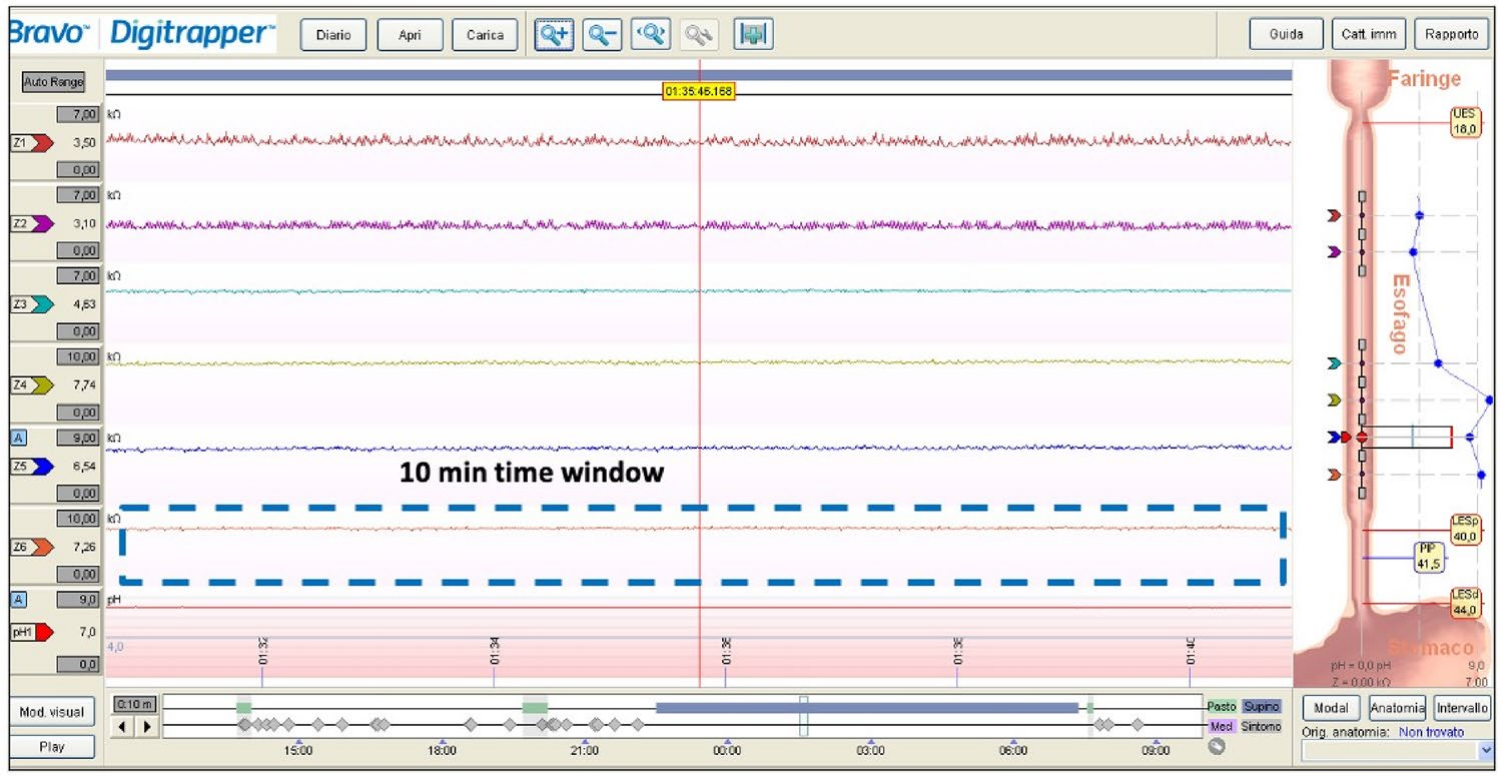
A 10-min window used to calculate the mean nocturnal baseline impedance at multichannel intraluminal pH-impedance monitoring

Esophageal baseline impedance values are inversely correlated with the AET, although other factors including eosinophilic esophagitis, esophageal body dilation, and esophageal motor disorders may alter baseline impedance independently of AET [56, 57]. In 2011, Farré et al. [58] demonstrated the correlation between esophageal transepithelial resistance and baseline impedance measurements, showing that baseline impedance values decreased after acid perfusion of the esophagus of healthy subjects. Since then, other investigators confirmed the relationship between baseline impedance and esophageal reflux burden, postulating the utility of baseline impedance for the evaluation of GERD [53, 56, 59, 60]. Accordingly, in 2014, Martinucci et al. [60] defined the MNBI as the mean impedance value calculated from MII-pH tracings in three 10-min windows during nighttime (around 1.00, 2.00, and 3.00 a.m.) (Fig. 1). The value of MNBI showed high correlation with impedance values calculated over a longer period of eight hours. These findings were recently confirmed in a study by Hoshikawa et al. [61]. In 2016, Frazzoni et al. [62] investigated the utility of MNBI at improving the diagnostic yield of MII-pH for the diagnosis of GERD. A cut-off value of MNBI < 2292 ohms showed an area under the curve (AUC) of 0.87 for the diagnosis of GERD; in addition, MNBI could segregate GERD subgroups with sensitivity of 91% and 86% for the diagnosis of erosive reflux disease and NERD, respectively. Additionally, MNBI showed potential to distinguish GERD subgroups, with lower values observed in patients with more severe mucosal damage, and increasingly higher values moving from erosive esophagitis, towards NERD and RH [63–65]. In contrast, MNBI values of patients with FH are comparable to those of healthy controls [59, 60]. Recently, Frazzoni et al. [66] validated the diagnostic yield of MNBI against AET thresholds according to Lyon Consensus. The authors found that an MNBI threshold value of 2000 ohms had AUC of 0.89 for the detection of patients with PPI-responsive NERD, with an odds ratio (OR) of 5.7 compared to AET of 4%.

With regard to therapeutic implications of MNBI, low MNBI values have been associated with PPI response in patients with normal or inconclusive MII-pH [63, 67, 68] (Table 1). Of note, MNBI showed a good performance at predicting response to treatment both in patients evaluated on- or off-PPI therapy [69]. Rengarajan et al. [67] investigated the utility of MNBI to predict response to medical anti-reflux treatment. The authors found that, both in patients with overtly abnormal (> 6%) or borderline AET (4–6%), a low MNBI identified patients who showed improvement with anti-reflux treatment. Recently, Gyawali et al. [70] investigated MII-pH metrics in patients with refractory GERD symptoms undergoing on-therapy reflux monitoring. The authors observed that, among 20 patients with AET > 4%, reflux episodes > 80, and MNBI < 1500 ohms, 85% improved with invasive GERD management.

**Table 1:** Performance of MNBI and PSPW at predicting response to proton pump inhibitors therapy

Author	Parameter (cut-off)	Sensitivity	Specificity	PPV	NPV	Odds ratio	AUC
de Bortoli 2015 [33]	MNBI (2446 Ω)	0.98	0.79	0.82	0.96		
Rengarajan 2020 [67]	MNBI (2292 Ω)	0.85	0.56	0.62	0.81		
Ribolsi 2020 [87]	MNBI (2292 Ω)	0.71	0.57	0.56	0.73		
	PSPW-I (61%)	0.75	0.59	0.58	0.76		
Frazzoni 2017 [64]	MNBI (2292 Ω)					3.586	0.742
	PSPW-I (61%)					12.449	0.795

*MNBI* mean nocturnal baseline impedance, *PSPW-I* post-reflux swallow-induced peristaltic wave index, *PPV* positive predictive value, *NPV* negative predictive value, *AUC* area under the curve

In conclusion, several studies showed that MNBI is useful to support the diagnosis of GERD, segregate GERD sub-types, and identify patients that will more likely respond to PPI treatment. Although the calculation of MNBI is reproducible and takes a few minutes [65, 71], it currently needs to be performed manually. However, AI tools for automated calculation of novel MII-pH metric are being developed [72]. In this regard, Rogers et al. developed an AI system which autonomously evaluated MII-pH tracings with an accuracy of 88.5% compared to human reviewers. Additionally, the ratio of upright baseline impedance divided by the recumbent baseline impedance (U:R AIBI ratio) could segregate responders to treatment from controls and nonresponders regardless of treatment status upon MII-pH recording. The U:R AIBI ratio at 5 cm above the LES performed better than AET in predicting response to medical therapy in those with conclusively abnormal AET as per the Lyon Consensus [72, 73]. Finally, MNBI is currently considered the most representative measure of baseline impedance [74] but novel techniques for the measurement of mucosal impedance have been recently proposed, including endoscopy ad hoc probes [75] and balloon catheter systems [76].

### Post-reflux swallow-induced peristaltic wave index

**Fig. 2:**
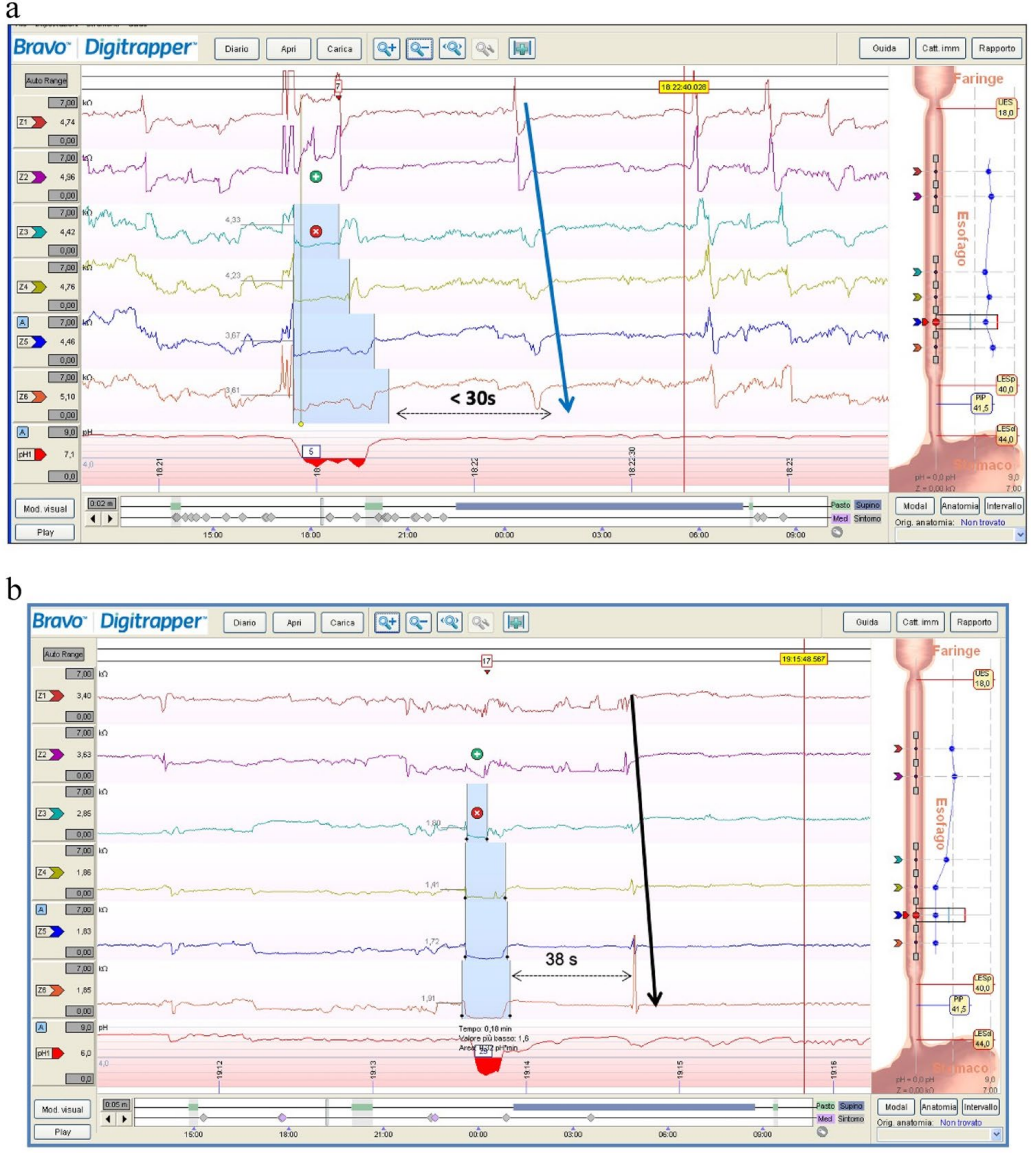
**a** Post-reflux swallow-induced peristaltic wave within 30 s after a reflux. This peristaltic wave should be included when calculating the PSPW index. **b** A peristaltic wave taking place 45 s after a reflux. This peristaltic wave should not be included when calculating the PSPW index

Once reflux occurs, the clearance of the refluxate from the esophageal lumen is triggered to protect the esophagus. Esophageal clearance is a biphasic phenomenon: the first component is a secondary peristaltic wave triggered by stretch receptors (volume clearance), while the second component is a primary peristaltic wave, elicited by an esophago-salivary vagal reflex, which delivers salivary bicarbonate and epidermal growth factor to the distal esophageal mucosa, providing chemical clearance and restoring a neutral pH [77]. Accordingly, PSPWs represent the mechanism of esophageal chemical clearance. At MII-pH, PSPWs are defined as an antegrade 50% drop in impedance relative to the pre-swallow baseline, originating in the most proximal impedance site, reaching all the distal impedance sites, and followed by at least a 50% return to the baseline in all the distal impedance sites [77, 78]. Of note, considering the latency period of salivary gland response to esophageal acidification (10–15 s) and a possible overlap with spontaneous swallowing (approximately 1 per minute), only PSPWs occurring within 30 s from the end of a reflux episode are believed to contribute to chemical clearance [77] (Fig. 2). Accordingly, a recent study concluded that a 30-s window for the assessment of PSPWs limits the risk of a casual association between reflux episodes and PSPW to around 30% [79]. At MII-pH analysis, PSPW-I is obtained by dividing the number of refluxes coupled with a PSPW by the number of total refluxes. Currently, the threshold of a normal PSPW-I is > 61% [62, 80, 81].

With regard to PSPW-I use in clinical practice, in a retrospective study on PPI-refractory GERD patients, PSPW-I was significantly lower in refractory esophagitis than in healed esophagitis and NERD (*p* = 0.003), and represented an estimate of the effectiveness of chemical clearance both for acidic and weakly acidic refluxes [82]. Accordingly, another study demonstrated a strong inverse correlation (*r* = − 0.889) between bolus clearance time and PSPW-I, confirming the importance of the mechanism for the clearance of the esophagus [33]. In addition, another study found a positive correlation (*r* = 0.623) between PSPW-I and baseline impedance values, providing proof of the role of chemical clearance for the maintenance of mucosal integrity [60]. The PSPW-I also showed a direct correlation (*r* = 0.626) with the degree of esophageal contractile reserve at HRM, which inversely correlates with AET. These findings support the hypothesis that acid exposure could affect the contractility of the esophageal muscle [83]. Recent evidence also showed that the presence of bile in the refluxate worsens heartburn severity, contributes to PPI-refractoriness, and is inversely correlated with PSPW (*r* = − 0.722), negatively affecting chemical clearance [84]. Of particular note, although a lower PSPW-I correlates with esophageal hypomotility, normal esophageal contractility does not appear to be essential for the generation of effective PSPWs. Additionally, the integrity of PSPWs seems to be more relevant than the contractile reserve for the clearance of the esophagus from refluxes [50]. In a prospective multicenter study, among MII-pH parameters, only PSPW-I was an independent risk factor for refractoriness to GERD treatment (OR 1.082, 95% CI 1.022–1.146, *p* = 0.007). Of note, when comparing on-PPI versus off-PPI MII-pH monitoring, the median value of PSPW-I did not change in PPI-refractory patients but increased significantly in PPI-responsive cases [81]. Similarly, PSPW-I has been associated with PPI-responsiveness and proved to be useful for the identification of patients requiring long-term PPIs [64, 85] (Table 1). In a study conducted on PPI-responsive GERD patients undergoing off-therapy MII-pH, PSPW-I had and AUC of 0.97 for the identification of patients with reflux disease. Compared to the use of conventional MII-pH parameters (i.e., AET, number of refluxes, and bolus exposure), PSPW-I showed higher sensitivity and overall accuracy for the diagnosis of GERD [62]. In another study, PSPW-I efficiently distinguished PPI-refractory NERD from FH during on-therapy MII-pH [65]. Additionally, PSPW-I was recently proposed as a useful metric to characterize RH when SI and SAP are inconclusive [86]. In this regard, in a retrospective study, PSPW-I and MNBI independently predicted the diagnosis of RH, with an AUC of 0.96 when assessed in combination. Accordingly, the assessment of PSPW-I and/or MNBI provided a significantly higher diagnostic yield compared to SI and SAP (62% vs 92%; *p* < 0.0001) [54].

In a recent study on the diagnostic yield of PSPW-I in patients with extra-esophageal GERD manifestations, Ribolsi et al. [87] found that the evaluation of MNBI and PSPW-I increased the diagnostic yield of impedance-pH compared to AET, SAP, and the presence of typical symptoms with a sensitivity of 75% and negative predictive value (NPV) of 76% for the diagnosis of GERD. Additionally, abnormal PSPW-I values were associated with a satisfactory response to acid-suppressive therapy in patients with extra-esophageal symptoms.

Finally, a retrospective study investigating reflux characteristics in 65 patients with BE demonstrated that PSPW-I could distinguish patients with and without incident dysplasia [88]. In particular, PSPW was significantly lower in the group that developed dysplasia than in the group that did not, both at the time of index (12% vs. 30%) and 3-year surveillance endoscopy (15% vs. 32%). Additionally, with a cut-off value of 26%, PSPW-I predicted neoplastic progression with accuracy, sensitivity, specificity, PPV, and NPV of 75%, 80%, 74%, 48%, and 93%, respectively [88].

## Conclusion

MII-pH monitoring provides the most comprehensive assessment of GERD. Indications for testing include treatment failure, diagnostic uncertainty, and preoperative assessment of GERD. PSPW-I and MNBI have been recently proposed by the Lyon Consensus as diagnostic modifiers when the diagnosis of GERD is unclear [27], although normal MII-pH monitoring thresholds have regional and system-related differences [80].

This review provided updated evidence on the utility of novel MII-pH metrics in different common clinical scenarios (Table 2). Although concerns have been raised on the time-consuming nature and possible variability in the calculation of novel MII-pH parameters [74, 89], MNBI and PSPW-I demonstrated high diagnostic yield for the categorization of GERD and several authors advocate their routine assessment [86, 87, 90]. In this regard, the recent Wingate Consensus provided expert recommendations for a standardized identification of PSPWs in clinical practice [91], and reproducible methods for the calculation of MNBI are available [60, 65, 71].

**Table 2:** Clinical use of MNBI and PSPW

Parameter	Clinical use	References
MNBI	Distinction between ERD and NERD	De Bortoli 2015 [63] Frazzoni 2016 [62] Frazzoni 2017 [64] Frazzoni 2017 [65]
	Prediction of treatment response in patients undergoing on- or off-therapy MII-pH monitoring	De Bortoli 2015 [63] Rengarajan 2020 [67] Ribolsi 2020 [87] Zhang 2020 [68] Frazzoni 2021 [66] Gyawali 2021[70] Rogers 2021 [73]
	Predictor of RH	Frazzoni 2017 [54]
PSPW index	Indicator of esophageal chemical clearance	de Bortoli 2015 [33] Frazzoni 2014 [82]
	Indicator of esophageal contraction reserve	Martinucci 2016 [83]
	Risk factor for refractoriness to GERD treatment	Frazzoni 2018 [81]
	Identification of patients requiring long-term PPI treatment	Frazzoni 2017 [64] Savarino 2011 [85]
	Distinction between PPI-refractory NERD from FH during on-therapy MII-pH	Frazzoni 2017 [65]
	Predictor of RH	Frazzoni 2017 [54]
	Predictor of response to acid-suppressive therapy in patients with extra-esophageal symptoms	Ribolsi 2020 [87]
	Predictor of dysplasia in patients with Barrett’s Esophagus	Frazzoni 2014 [88]

*ERD* erosive reflux disease, *NERD* non-erosive reflux disease, *GERD* gastro-esophageal reflux disease, *PPI* proton pump inhibitors, *MII-pH* multichannel intraluminal pH-impedance, *MNBI* mean nocturnal baseline impedance, *PSPW* post-reflux swallow-induced peristaltic wave, *FH* functional heartburn, *RH* reflux hypersensitivity

Based on currently available evidence, we strongly support the evaluation of PSPW index and MNBI as part of the standard assessment of MII-pH tracings for the evaluation of GERD, especially in patients with endoscopy-negative heartburn.

## Declarations

### Guarantor of the article

Prof Nicola de Bortoli.
